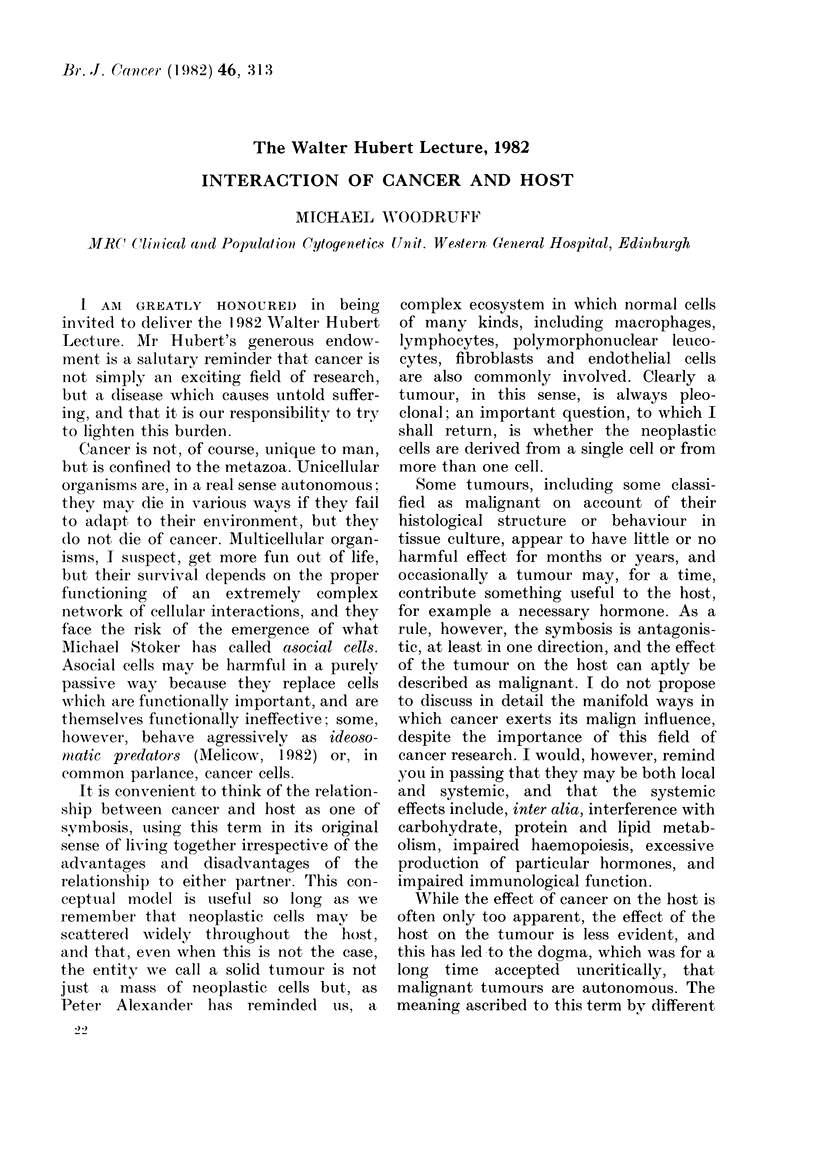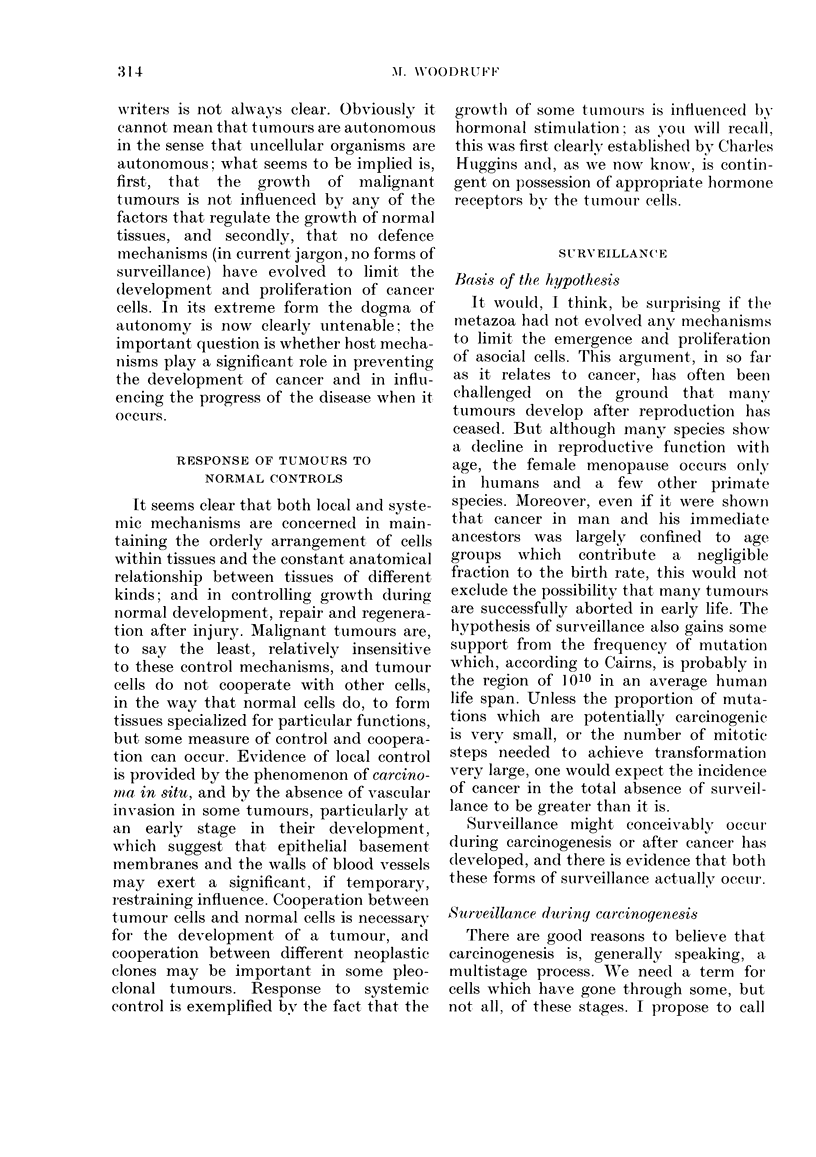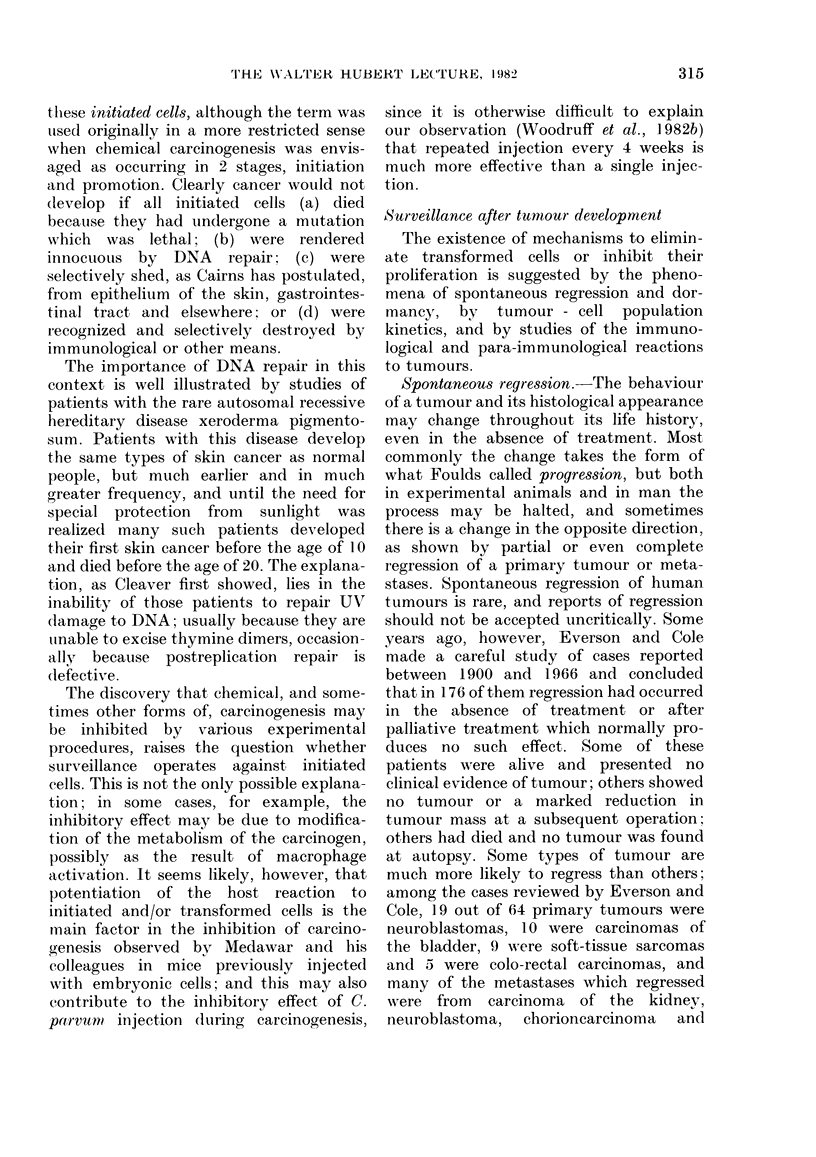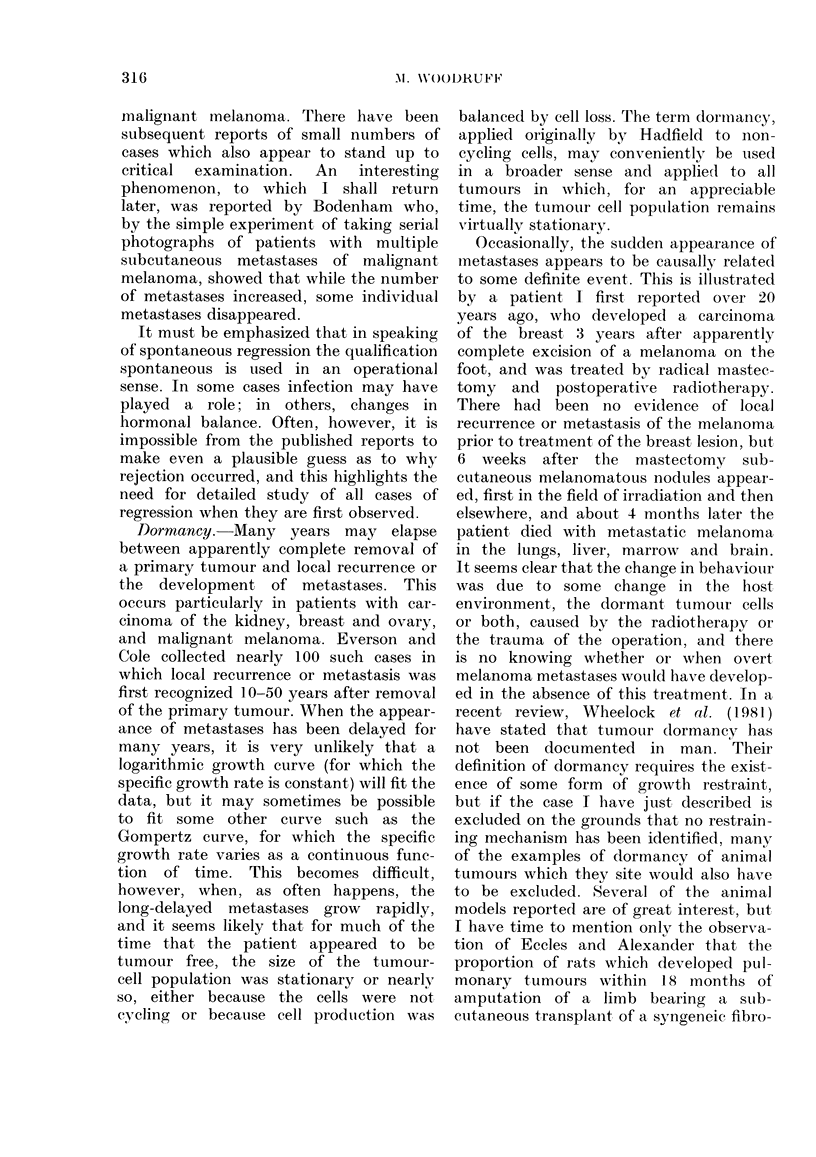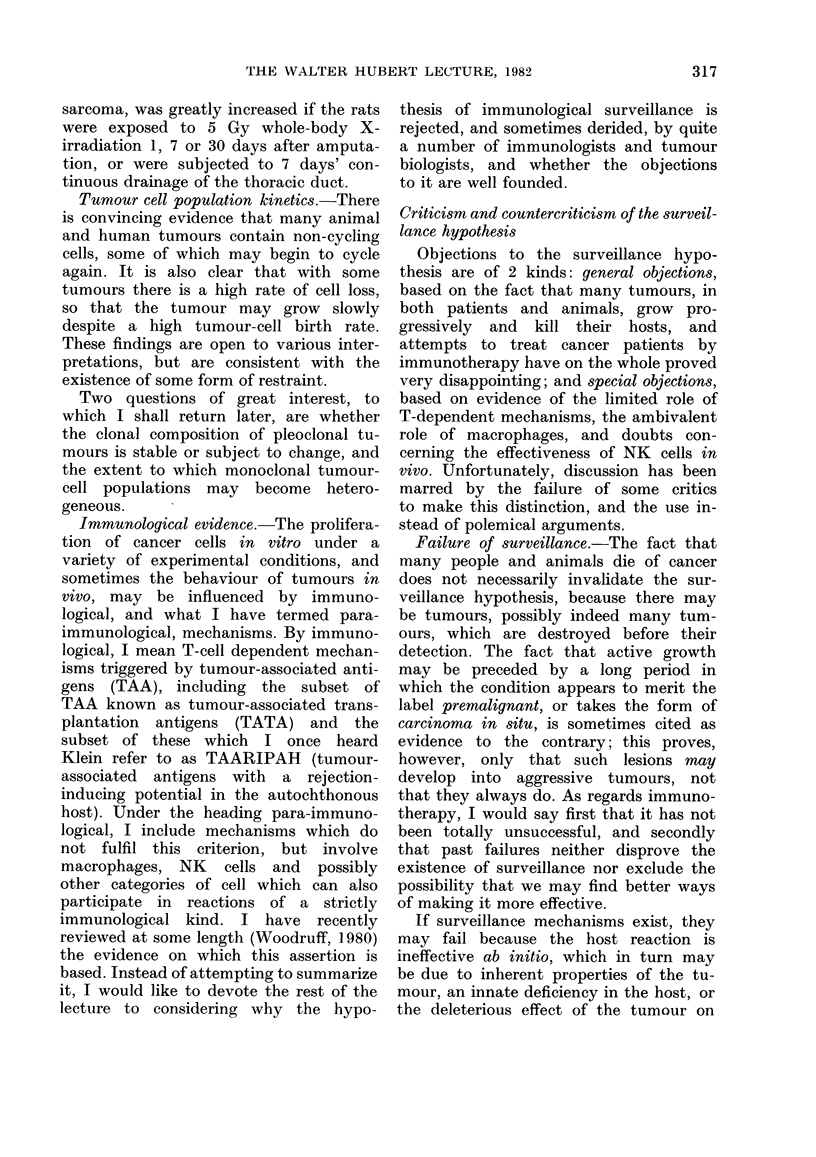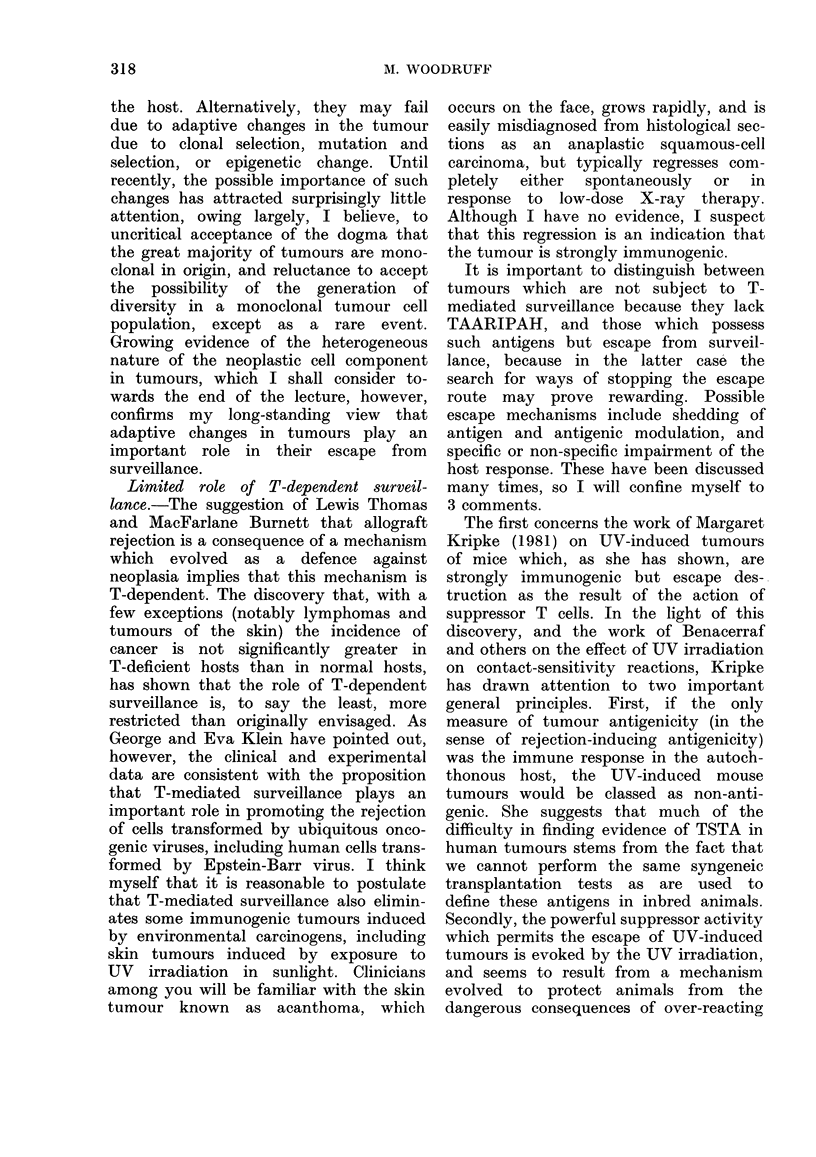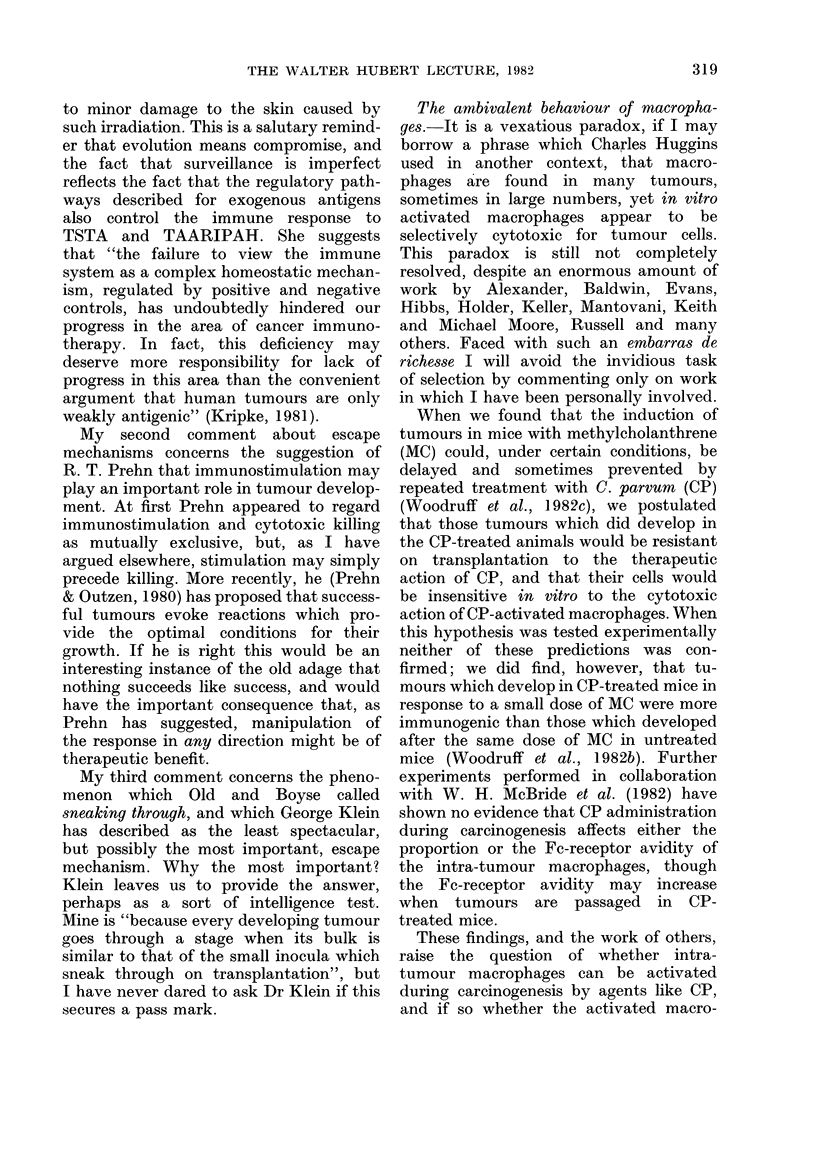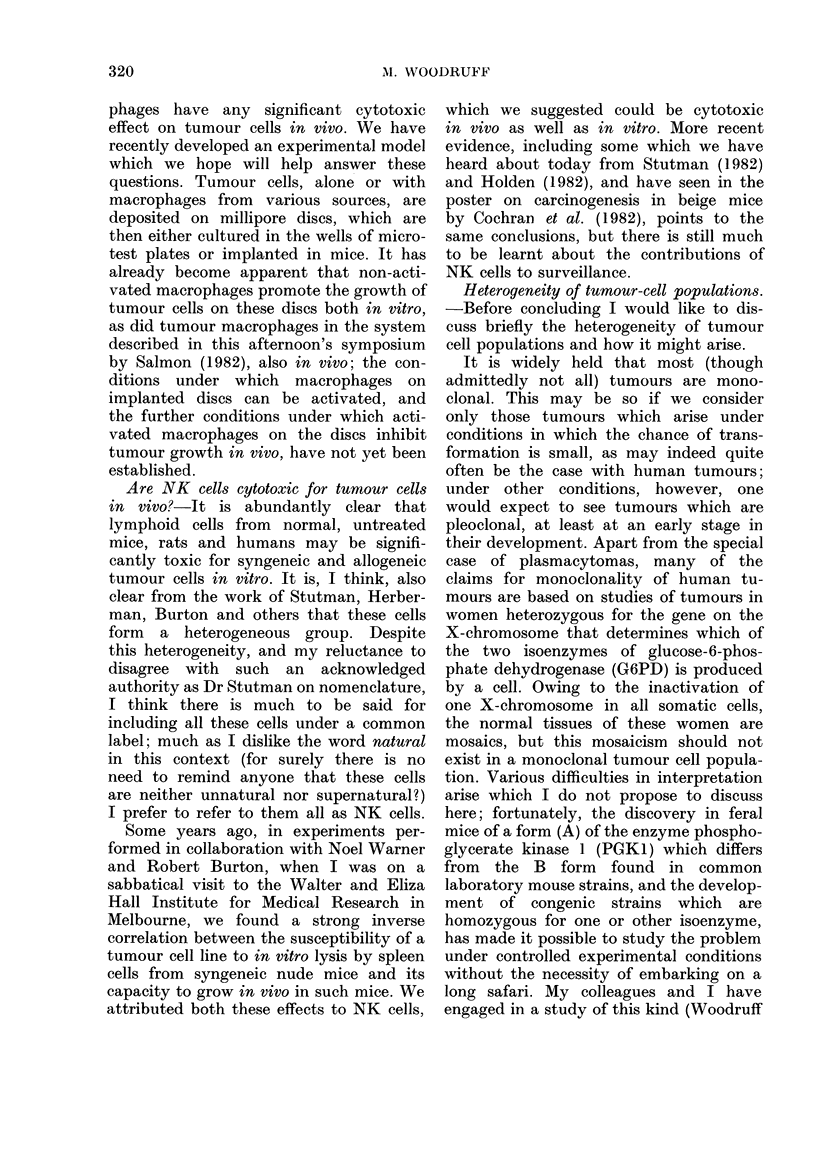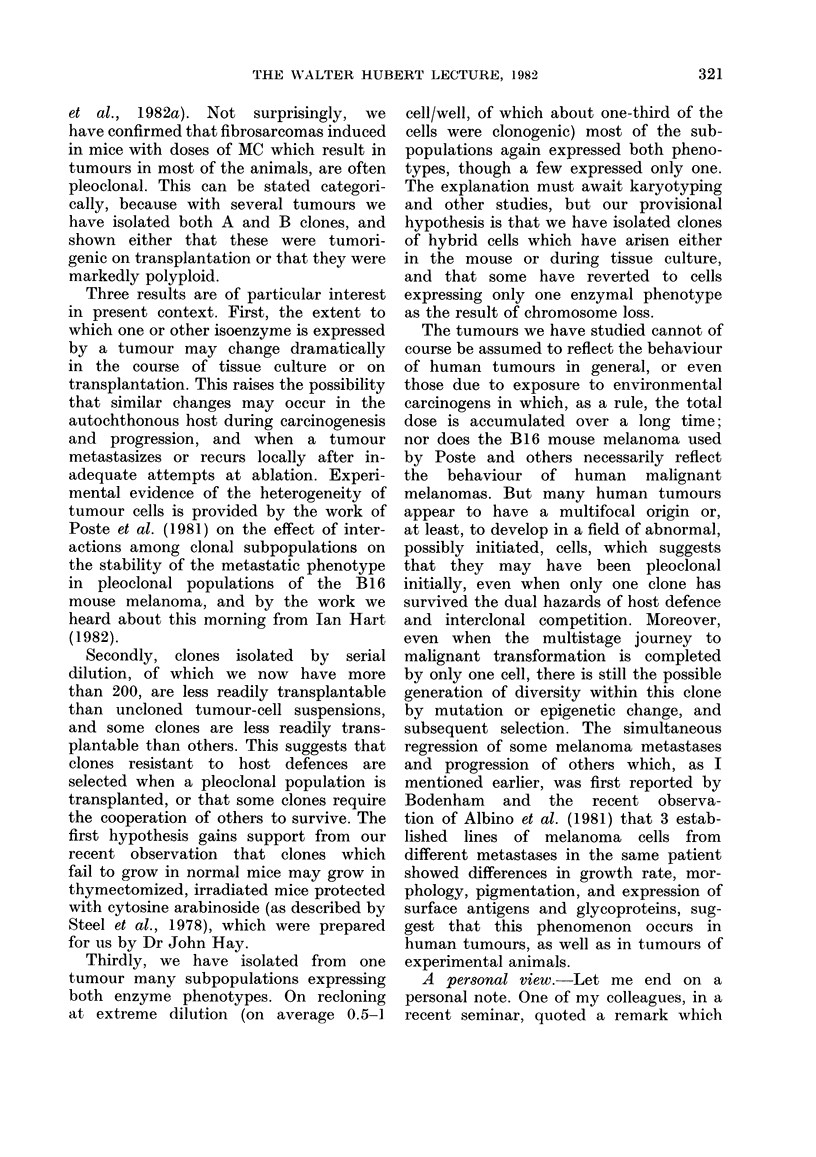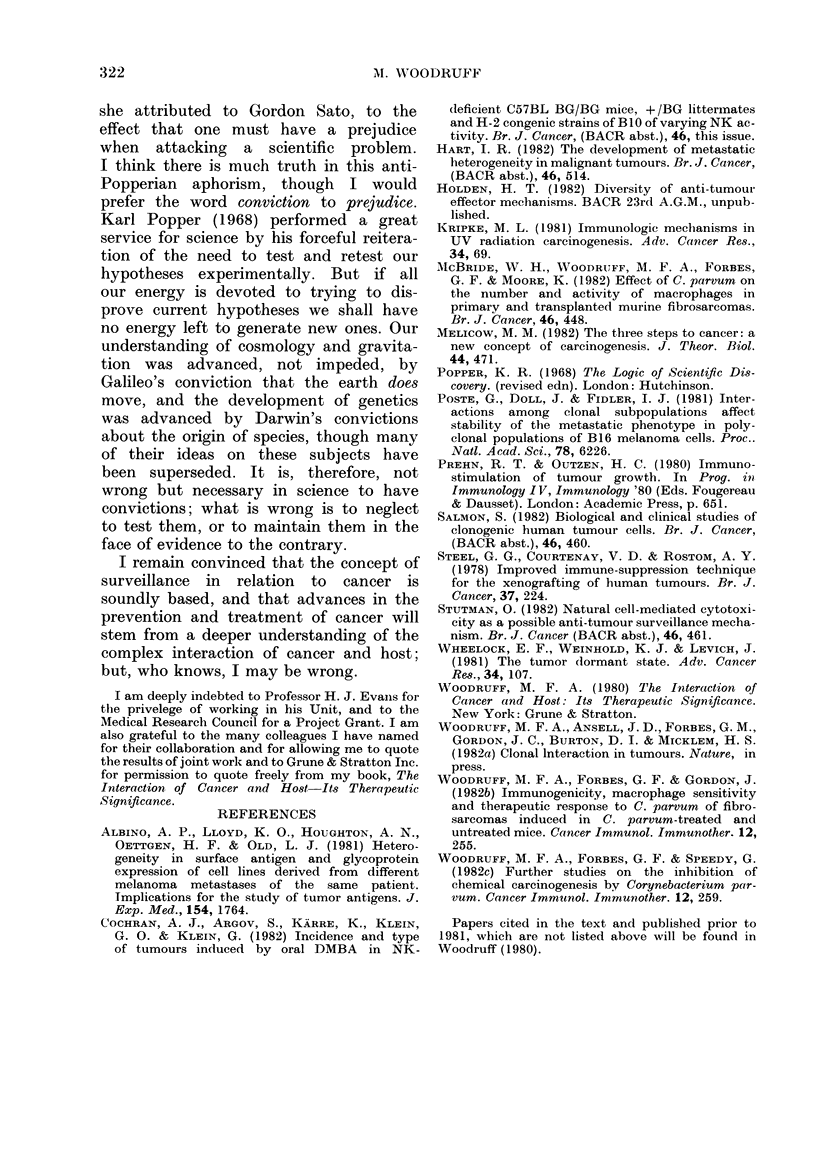# The Walter Hubert Lecture, 1982. Interaction of cancer and host.

**DOI:** 10.1038/bjc.1982.206

**Published:** 1982-09

**Authors:** M. Woodruff


Br. J. Cancer (1982) 46, 313

### The Walter Hubert Lecture, 1982

### INTERACTION OF CANCER AND HOST

MICHAEL WOODRUFF

*MRC Clinical and Population Cytogenetics Unit, Western General Hospital, Edinburgh*

I AM GREATLY HONOURED in being invited to deliver the 1982 Walter Hubert Lecture. Mr Hubert's generous endowment is a salutary reminder that cancer is not simply an exciting field of research, but a disease which causes untold suffering, and that it is our responsibility to try to lighten this burden.

Cancer is not, of course, unique to man, but is confined to the metazoa. Unicellular organisms are, in a real sense autonomous; they may die in various ways if they fail to adapt to their environment, but they do not die of cancer. Multicellular organisms, I suspect, get more fun out of life, but their survival depends on the proper functioning of an extremely complex network of cellular interactions, and they face the risk of the emergence of what Michael Stoker has called *asocial cells*. Asocial cells may be harmful in a purely passive way because they replace cells which are functionally important, and are themselves functionally ineffective; some, however, behave agressively as *ideosomatic predators* (Melicow, 1982) or, in common parlance, cancer cells.

It is convenient to think of the relationship between cancer and host as one of symbosis, using this term in its original sense of living together irrespective of the advantages and disadvantages of the relationship to either partner. This conceptual model is useful so long as we remember that neoplastic cells may be scattered widely throughout the host, and that, even when this is not the case, the entity we call a solid tumour is not just a mass of neoplastic cells but, as Peter Alexander has reminded us, a complex ecosystem in which normal cells of many kinds, including macrophages, lymphocytes, polymorphonuclear leucocytes, fibroblasts and endothelial cells are also commonly involved. Clearly a tumour, in this sense, is always pleoclonal; an important question, to which I shall return, is whether the neoplastic cells are derived from a single cell or from more than one cell.

Some tumours, including some classified as malignant on account of their histological structure or behaviour in tissue culture, appear to have little or no harmful effect for months or years, and occasionally a tumour may, for a time, contribute something useful to the host, for example a necessary hormone. As a rule, however, the symbosis is antagonistic, at least in one direction, and the effect of the tumour on the host can aptly be described as malignant. I do not propose to discuss in detail the manifold ways in which cancer exerts its malign influence, despite the importance of this field of cancer research. I would, however, remind you in passing that they may be both local and systemic, and that the systemic effects include, *inter alia*, interference with carbohydrate, protein and lipid metabolism, impaired haemopoiesis, excessive production of particular hormones, and impaired immunological function.

While the effect of cancer on the host is often only too apparent, the effect of the host on the tumour is less evident, and this has led to the dogma, which was for a long time accepted uncritically, that malignant tumours are autonomous. The meaning ascribed to this term by different writers is not always clear. Obviously it cannot mean that tumours are autonomous in the sense that uncellular organisms are autonomous; what seems to be implied is, first, that the growth of malignant tumours is not influenced by any of the factors that regulate the growth of normal tissues, and secondly, that no defence mechanisms (in current jargon, no forms of surveillance) have evolved to limit the development and proliferation of cancer cells. In its extreme form the dogma of autonomy is now clearly untenable; the important question is whether host mechanisms play a significant role in preventing the development of cancer and in influencing the progress of the disease when it occurs.

### RESPONSE OF TUMOURS TO NORMAL CONTROLS

It seems clear that both local and systemic mechanisms are concerned in maintaining the orderly arrangement of cells within tissues and the constant anatomical relationship between tissues of different kinds; and in controlling growth during normal development, repair and regeneration after injury. Malignant tumours are, to say the least, relatively insensitive to these control mechanisms, and tumour cells do not cooperate with other cells, in the way that normal cells do, to form tissues specialized for particular functions, but some measure of control and cooperation can occur. Evidence of local control is provided by the phenomenon of *carcinoma in situ*, and by the absence of vascular invasion in some tumours, particularly at an early stage in their development, which suggest that epithelial basement membranes and the walls of blood vessels may exert a significant, if temporary, restraining influence. Cooperation between tumour cells and normal cells is necessary for the development of a tumour, and cooperation between different neoplastic clones may be important in some pleoclonal tumours. Response to systemic control is exemplified by the fact that the growth of some tumours is influenced by hormonal stimulation; as you will recall, this was first clearly established by Charles Huggins and, as we now know, is contingent on possession of appropriate hormone receptors by the tumour cells.

### SURVEILLANCE

### *Basis of the hypothesis*

It would, I think, be surprising if the metazoa had not evolved any mechanisms to limit the emergence and proliferation of asocial cells. This argument, in so far as it relates to cancer, has often been challenged on the ground that many tumours develop after reproduction has ceased. But although many species show a decline in reproductive function with age, the female menopause occurs only in humans and a few other primate species. Moreover, even if it were shown that cancer in man and his immediate ancestors was largely confined to age groups which contribute a negligible fraction to the birth rate, this would not exclude the possibility that many tumours are successfully aborted in early life. The hypothesis of surveillance also gains some support from the frequency of mutation which, according to Cairns, is probably in the region of $10^{10}$ in an average human life span. Unless the proportion of mutations which are potentially carcinogenic is very small, or the number of mitotic steps needed to achieve transformation very large, one would expect the incidence of cancer in the total absence of surveillance to be greater than it is.

Surveillance might conceivably occur during carcinogenesis or after cancer has developed, and there is evidence that both these forms of surveillance actually occur.

### *Surveillance during carcinogenesis*

There are good reasons to believe that carcinogenesis is, generally speaking, a multistage process. We need a term for cells which have gone through some, but not all, of these stages. I propose to call these *initiated cells*, although the term was used originally in a more restricted sense when chemical carcinogenesis was envisaged as occurring in 2 stages, initiation and promotion. Clearly cancer would not develop if all initiated cells (a) died because they had undergone a mutation which was lethal; (b) were rendered innocuous by DNA repair; (c) were selectively shed, as Cairns has postulated, from epithelium of the skin, gastrointestinal tract and elsewhere; or (d) were recognized and selectively destroyed by immunological or other means.

The importance of DNA repair in this context is well illustrated by studies of patients with the rare autosomal recessive hereditary disease xeroderma pigmentosum. Patients with this disease develop the same types of skin cancer as normal people, but much earlier and in much greater frequency, and until the need for special protection from sunlight was realized many such patients developed their first skin cancer before the age of 10 and died before the age of 20. The explanation, as Cleaver first showed, lies in the inability of those patients to repair UV damage to DNA; usually because they are unable to excise thymine dimers, occasionally because postreplication repair is defective.

The discovery that chemical, and sometimes other forms of, carcinogenesis may be inhibited by various experimental procedures, raises the question whether surveillance operates against initiated cells. This is not the only possible explanation; in some cases, for example, the inhibitory effect may be due to modification of the metabolism of the carcinogen, possibly as the result of macrophage activation. It seems likely, however, that potentiation of the host reaction to initiated and/or transformed cells is the main factor in the inhibition of carcinogenesis observed by Medawar and his colleagues in mice previously injected with embryonic cells; and this may also contribute to the inhibitory effect of *C. parvum* injection during carcinogenesis, since it is otherwise difficult to explain our observation (Woodruff *et al.*, 1982*b*) that repeated injection every 4 weeks is much more effective than a single injection.

*Surveillance after tumour development*

The existence of mechanisms to eliminate transformed cells or inhibit their proliferation is suggested by the phenomena of spontaneous regression and dormancy, by tumour - cell population kinetics, and by studies of the immunological and para-immunological reactions to tumours.

*Spontaneous regression.*—The behaviour of a tumour and its histological appearance may change throughout its life history, even in the absence of treatment. Most commonly the change takes the form of what Foulds called *progression*, but both in experimental animals and in man the process may be halted, and sometimes there is a change in the opposite direction, as shown by partial or even complete regression of a primary tumour or metastases. Spontaneous regression of human tumours is rare, and reports of regression should not be accepted uncritically. Some years ago, however, Everson and Cole made a careful study of cases reported between 1900 and 1966 and concluded that in 176 of them regression had occurred in the absence of treatment or after palliative treatment which normally produces no such effect. Some of these patients were alive and presented no clinical evidence of tumour; others showed no tumour or a marked reduction in tumour mass at a subsequent operation; others had died and no tumour was found at autopsy. Some types of tumour are much more likely to regress than others; among the cases reviewed by Everson and Cole, 19 out of 64 primary tumours were neuroblastomas, 10 were carcinomas of the bladder, 9 were soft-tissue sarcomas and 5 were colo-rectal carcinomas, and many of the metastases which regressed were from carcinoma of the kidney, neuroblastoma, chorioncarcinoma and malignant melanoma. There have been subsequent reports of small numbers of cases which also appear to stand up to critical examination. An interesting phenomenon, to which I shall return later, was reported by Bodenham who, by the simple experiment of taking serial photographs of patients with multiple subcutaneous metastases of malignant melanoma, showed that while the number of metastases increased, some individual metastases disappeared.

It must be emphasized that in speaking of spontaneous regression the qualification spontaneous is used in an operational sense. In some cases infection may have played a role; in others, changes in hormonal balance. Often, however, it is impossible from the published reports to make even a plausible guess as to why rejection occurred, and this highlights the need for detailed study of all cases of regression when they are first observed.

*Dormancy.*—Many years may elapse between apparently complete removal of a primary tumour and local recurrence or the development of metastases. This occurs particularly in patients with carcinoma of the kidney, breast and ovary, and malignant melanoma. Everson and Cole collected nearly 100 such cases in which local recurrence or metastasis was first recognized 10–50 years after removal of the primary tumour. When the appearance of metastases has been delayed for many years, it is very unlikely that a logarithmic growth curve (for which the specific growth rate is constant) will fit the data, but it may sometimes be possible to fit some other curve such as the Gompertz curve, for which the specific growth rate varies as a continuous function of time. This becomes difficult, however, when, as often happens, the long-delayed metastases grow rapidly, and it seems likely that for much of the time that the patient appeared to be tumour free, the size of the tumour-cell population was stationary or nearly so, either because the cells were not cycling or because cell production was balanced by cell loss. The term dormancy, applied originally by Hadfield to non-cycling cells, may conveniently be used in a broader sense and applied to all tumours in which, for an appreciable time, the tumour cell population remains virtually stationary.

Occasionally, the sudden appearance of metastases appears to be causally related to some definite event. This is illustrated by a patient I first reported over 20 years ago, who developed a carcinoma of the breast 3 years after apparently complete excision of a melanoma on the foot, and was treated by radical mastectomy and postoperative radiotherapy. There had been no evidence of local recurrence or metastasis of the melanoma prior to treatment of the breast lesion, but 6 weeks after the mastectomy subcutaneous melanomatous nodules appeared, first in the field of irradiation and then elsewhere, and about 4 months later the patient died with metastatic melanoma in the lungs, liver, marrow and brain. It seems clear that the change in behaviour was due to some change in the host environment, the dormant tumour cells or both, caused by the radiotherapy or the trauma of the operation, and there is no knowing whether or when overt melanoma metastases would have developed in the absence of this treatment. In a recent review, Wheelock *et al.* (1981) have stated that tumour dormancy has not been documented in man. Their definition of dormancy requires the existence of some form of growth restraint, but if the case I have just described is excluded on the grounds that no restraining mechanism has been identified, many of the examples of dormancy of animal tumours which they site would also have to be excluded. Several of the animal models reported are of great interest, but I have time to mention only the observation of Eccles and Alexander that the proportion of rats which developed pulmonary tumours within 18 months of amputation of a limb bearing a subcutaneous transplant of a syngeneic fibrosarcoma, was greatly increased if the rats were exposed to 5 Gy whole-body X-irradiation 1, 7 or 30 days after amputation, or were subjected to 7 days' continuous drainage of the thoracic duct.

*Tumour cell population kinetics.*—There is convincing evidence that many animal and human tumours contain non-cycling cells, some of which may begin to cycle again. It is also clear that with some tumours there is a high rate of cell loss, so that the tumour may grow slowly despite a high tumour-cell birth rate. These findings are open to various interpretations, but are consistent with the existence of some form of restraint.

Two questions of great interest, to which I shall return later, are whether the clonal composition of pleoclonal tumours is stable or subject to change, and the extent to which monoclonal tumour-cell populations may become heterogeneous.

*Immunological evidence.*—The proliferation of cancer cells *in vitro* under a variety of experimental conditions, and sometimes the behaviour of tumours *in vivo*, may be influenced by immunological, and what I have termed para-immunological, mechanisms. By immunological, I mean T-cell dependent mechanisms triggered by tumour-associated antigens (TAA), including the subset of TAA known as tumour-associated transplantation antigens (TATA) and the subset of these which I once heard Klein refer to as TAARIPAH (tumour-associated antigens with a rejection-inducing potential in the autochthonous host). Under the heading para-immunological, I include mechanisms which do not fulfil this criterion, but involve macrophages, NK cells and possibly other categories of cell which can also participate in reactions of a strictly immunological kind. I have recently reviewed at some length (Woodruff, 1980) the evidence on which this assertion is based. Instead of attempting to summarize it, I would like to devote the rest of the lecture to considering why the hypothesis of immunological surveillance is rejected, and sometimes derided, by quite a number of immunologists and tumour biologists, and whether the objections to it are well founded.

### *Criticism and countercriticism of the surveillance hypothesis*

Objections to the surveillance hypothesis are of 2 kinds: *general objections*, based on the fact that many tumours, in both patients and animals, grow progressively and kill their hosts, and attempts to treat cancer patients by immunotherapy have on the whole proved very disappointing; and *special objections*, based on evidence of the limited role of T-dependent mechanisms, the ambivalent role of macrophages, and doubts concerning the effectiveness of NK cells *in vivo*. Unfortunately, discussion has been marred by the failure of some critics to make this distinction, and the use instead of polemical arguments.

*Failure of surveillance.*—The fact that many people and animals die of cancer does not necessarily invalidate the surveillance hypothesis, because there may be tumours, possibly indeed many tumours, which are destroyed before their detection. The fact that active growth may be preceded by a long period in which the condition appears to merit the label *premalignant*, or takes the form of *carcinoma in situ*, is sometimes cited as evidence to the contrary; this proves, however, only that such lesions *may* develop into aggressive tumours, not that they always do. As regards immunotherapy, I would say first that it has not been totally unsuccessful, and secondly that past failures neither disprove the existence of surveillance nor exclude the possibility that we may find better ways of making it more effective.

If surveillance mechanisms exist, they may fail because the host reaction is ineffective *ab initio*, which in turn may be due to inherent properties of the tumour, an innate deficiency in the host, or the deleterious effect of the tumour on the host. Alternatively, they may fail due to adaptive changes in the tumour due to clonal selection, mutation and selection, or epigenetic change. Until recently, the possible importance of such changes has attracted surprisingly little attention, owing largely, I believe, to uncritical acceptance of the dogma that the great majority of tumours are monoclonal in origin, and reluctance to accept the possibility of the generation of diversity in a monoclonal tumour cell population, except as a rare event. Growing evidence of the heterogeneous nature of the neoplastic cell component in tumours, which I shall consider towards the end of the lecture, however, confirms my long-standing view that adaptive changes in tumours play an important role in their escape from surveillance.

*Limited role of T-dependent surveillance.*—The suggestion of Lewis Thomas and MacFarlane Burnett that allograft rejection is a consequence of a mechanism which evolved as a defence against neoplasia implies that this mechanism is T-dependent. The discovery that, with a few exceptions (notably lymphomas and tumours of the skin) the incidence of cancer is not significantly greater in T-deficient hosts than in normal hosts, has shown that the role of T-dependent surveillance is, to say the least, more restricted than originally envisaged. As George and Eva Klein have pointed out, however, the clinical and experimental data are consistent with the proposition that T-mediated surveillance plays an important role in promoting the rejection of cells transformed by ubiquitous oncogenic viruses, including human cells transformed by Epstein-Barr virus. I think myself that it is reasonable to postulate that T-mediated surveillance also eliminates some immunogenic tumours induced by environmental carcinogens, including skin tumours induced by exposure to UV irradiation in sunlight. Clinicians among you will be familiar with the skin tumour known as acanthoma, which occurs on the face, grows rapidly, and is easily misdiagnosed from histological sections as an anaplastic squamous-cell carcinoma, but typically regresses completely either spontaneously or in response to low-dose X-ray therapy. Although I have no evidence, I suspect that this regression is an indication that the tumour is strongly immunogenic.

It is important to distinguish between tumours which are not subject to T-mediated surveillance because they lack TAARIPAH, and those which possess such antigens but escape from surveillance, because in the latter case the search for ways of stopping the escape route may prove rewarding. Possible escape mechanisms include shedding of antigen and antigenic modulation, and specific or non-specific impairment of the host response. These have been discussed many times, so I will confine myself to 3 comments.

The first concerns the work of Margaret Kripke (1981) on UV-induced tumours of mice which, as she has shown, are strongly immunogenic but escape destruction as the result of the action of suppressor T cells. In the light of this discovery, and the work of Benacerraf and others on the effect of UV irradiation on contact-sensitivity reactions, Kripke has drawn attention to two important general principles. First, if the only measure of tumour antigenicity (in the sense of rejection-inducing antigenicity) was the immune response in the autochthonous host, the UV-induced mouse tumours would be classed as non-antigenic. She suggests that much of the difficulty in finding evidence of TSTA in human tumours stems from the fact that we cannot perform the same syngeneic transplantation tests as are used to define these antigens in inbred animals. Secondly, the powerful suppressor activity which permits the escape of UV-induced tumours is evoked by the UV irradiation, and seems to result from a mechanism evolved to protect animals from the dangerous consequences of over-reacting to minor damage to the skin caused by such irradiation. This is a salutary reminder that evolution means compromise, and the fact that surveillance is imperfect reflects the fact that the regulatory pathways described for exogenous antigens also control the immune response to TSTA and TAARIPAH. She suggests that "the failure to view the immune system as a complex homeostatic mechanism, regulated by positive and negative controls, has undoubtedly hindered our progress in the area of cancer immunotherapy. In fact, this deficiency may deserve more responsibility for lack of progress in this area than the convenient argument that human tumours are only weakly antigenic" (Kripke, 1981).

My second comment about escape mechanisms concerns the suggestion of R. T. Prehn that immunostimulation may play an important role in tumour development. At first Prehn appeared to regard immunostimulation and cytotoxic killing as mutually exclusive, but, as I have argued elsewhere, stimulation may simply precede killing. More recently, he (Prehn & Outzen, 1980) has proposed that successful tumours evoke reactions which provide the optimal conditions for their growth. If he is right this would be an interesting instance of the old adage that nothing succeeds like success, and would have the important consequence that, as Prehn has suggested, manipulation of the response in *any* direction might be of therapeutic benefit.

My third comment concerns the phenomenon which Old and Boyse called *sneaking through*, and which George Klein has described as the least spectacular, but possibly the most important, escape mechanism. Why the most important? Klein leaves us to provide the answer, perhaps as a sort of intelligence test. Mine is "because every developing tumour goes through a stage when its bulk is similar to that of the small inocula which sneak through on transplantation", but I have never dared to ask Dr Klein if this secures a pass mark.

*The ambivalent behaviour of macrophages.*—It is a vexatious paradox, if I may borrow a phrase which Charles Huggins used in another context, that macrophages are found in many tumours, sometimes in large numbers, yet *in vitro* activated macrophages appear to be selectively cytotoxic for tumour cells. This paradox is still not completely resolved, despite an enormous amount of work by Alexander, Baldwin, Evans, Hibbs, Holder, Keller, Mantovani, Keith and Michael Moore, Russell and many others. Faced with such an *embarras de richesse* I will avoid the invidious task of selection by commenting only on work in which I have been personally involved.

When we found that the induction of tumours in mice with methylcholanthrene (MC) could, under certain conditions, be delayed and sometimes prevented by repeated treatment with *C. parvum* (CP) (Woodruff *et al.*, 1982*c*), we postulated that those tumours which did develop in the CP-treated animals would be resistant on transplantation to the therapeutic action of CP, and that their cells would be insensitive *in vitro* to the cytotoxic action of CP-activated macrophages. When this hypothesis was tested experimentally neither of these predictions was confirmed; we did find, however, that tumours which develop in CP-treated mice in response to a small dose of MC were more immunogenic than those which developed after the same dose of MC in untreated mice (Woodruff *et al.*, 1982*b*). Further experiments performed in collaboration with W. H. McBride *et al.* (1982) have shown no evidence that CP administration during carcinogenesis affects either the proportion or the Fc-receptor avidity of the intra-tumour macrophages, though the Fc-receptor avidity may increase when tumours are passaged in CP-treated mice.

These findings, and the work of others, raise the question of whether intra-tumour macrophages can be activated during carcinogenesis by agents like CP, and if so whether the activated macrophages have any significant cytotoxic effect on tumour cells *in vivo*. We have recently developed an experimental model which we hope will help answer these questions. Tumour cells, alone or with macrophages from various sources, are deposited on millipore discs, which are then either cultured in the wells of microtest plates or implanted in mice. It has already become apparent that non-activated macrophages promote the growth of tumour cells on these discs both *in vitro*, as did tumour macrophages in the system described in this afternoon's symposium by Salmon (1982), also *in vivo*; the conditions under which macrophages on implanted discs can be activated, and the further conditions under which activated macrophages on the discs inhibit tumour growth *in vivo*, have not yet been established.

*Are NK cells cytotoxic for tumour cells in vivo?*—It is abundantly clear that lymphoid cells from normal, untreated mice, rats and humans may be significantly toxic for syngeneic and allogeneic tumour cells *in vitro*. It is, I think, also clear from the work of Stutman, Herberman, Burton and others that these cells form a heterogeneous group. Despite this heterogeneity, and my reluctance to disagree with such an acknowledged authority as Dr Stutman on nomenclature, I think there is much to be said for including all these cells under a common label; much as I dislike the word *natural* in this context (for surely there is no need to remind anyone that these cells are neither unnatural nor supernatural?) I prefer to refer to them all as NK cells.

Some years ago, in experiments performed in collaboration with Noel Warner and Robert Burton, when I was on a sabbatical visit to the Walter and Eliza Hall Institute for Medical Research in Melbourne, we found a strong inverse correlation between the susceptibility of a tumour cell line to *in vitro* lysis by spleen cells from syngeneic nude mice and its capacity to grow *in vivo* in such mice. We attributed both these effects to NK cells, which we suggested could be cytotoxic *in vivo* as well as *in vitro*. More recent evidence, including some which we have heard about today from Stutman (1982) and Holden (1982), and have seen in the poster on carcinogenesis in beige mice by Cochran *et al.* (1982), points to the same conclusions, but there is still much to be learnt about the contributions of NK cells to surveillance.

*Heterogeneity of tumour-cell populations.*—Before concluding I would like to discuss briefly the heterogeneity of tumour cell populations and how it might arise.

It is widely held that most (though admittedly not all) tumours are monoclonal. This may be so if we consider only those tumours which arise under conditions in which the chance of transformation is small, as may indeed quite often be the case with human tumours; under other conditions, however, one would expect to see tumours which are pleoclonal, at least at an early stage in their development. Apart from the special case of plasmacytomas, many of the claims for monoclonality of human tumours are based on studies of tumours in women heterozygous for the gene on the X-chromosome that determines which of the two isoenzymes of glucose-6-phosphate dehydrogenase (G6PD) is produced by a cell. Owing to the inactivation of one X-chromosome in all somatic cells, the normal tissues of these women are mosaics, but this mosaicism should not exist in a monoclonal tumour cell population. Various difficulties in interpretation arise which I do not propose to discuss here; fortunately, the discovery in feral mice of a form (A) of the enzyme phosphoglycerate kinase 1 (PGK1) which differs from the B form found in common laboratory mouse strains, and the development of congenic strains which are homozygous for one or other isoenzyme, has made it possible to study the problem under controlled experimental conditions without the necessity of embarking on a long safari. My colleagues and I have engaged in a study of this kind (Woodruff

*et al.*, 1982*a*). Not surprisingly, we have confirmed that fibrosarcomas induced in mice with doses of MC which result in tumours in most of the animals, are often pleoclonal. This can be stated categorically, because with several tumours we have isolated both A and B clones, and shown either that these were tumorigenic on transplantation or that they were markedly polyploid.

Three results are of particular interest in present context. First, the extent to which one or other isoenzyme is expressed by a tumour may change dramatically in the course of tissue culture or on transplantation. This raises the possibility that similar changes may occur in the autochthonous host during carcinogenesis and progression, and when a tumour metastasizes or recurs locally after inadequate attempts at ablation. Experimental evidence of the heterogeneity of tumour cells is provided by the work of Poste *et al.* (1981) on the effect of interactions among clonal subpopulations on the stability of the metastatic phenotype in pleoclonal populations of the B16 mouse melanoma, and by the work we heard about this morning from Ian Hart (1982).

Secondly, clones isolated by serial dilution, of which we now have more than 200, are less readily transplantable than uncloned tumour-cell suspensions, and some clones are less readily transplantable than others. This suggests that clones resistant to host defences are selected when a pleoclonal population is transplanted, or that some clones require the cooperation of others to survive. The first hypothesis gains support from our recent observation that clones which fail to grow in normal mice may grow in thymectomized, irradiated mice protected with cytosine arabinoside (as described by Steel *et al.*, 1978), which were prepared for us by Dr John Hay.

Thirdly, we have isolated from one tumour many subpopulations expressing both enzyme phenotypes. On recloning at extreme dilution (on average 0.5–1 cell/well, of which about one-third of the cells were clonogenic) most of the subpopulations again expressed both phenotypes, though a few expressed only one. The explanation must await karyotyping and other studies, but our provisional hypothesis is that we have isolated clones of hybrid cells which have arisen either in the mouse or during tissue culture, and that some have reverted to cells expressing only one enzymal phenotype as the result of chromosome loss.

The tumours we have studied cannot of course be assumed to reflect the behaviour of human tumours in general, or even those due to exposure to environmental carcinogens in which, as a rule, the total dose is accumulated over a long time; nor does the B16 mouse melanoma used by Poste and others necessarily reflect the behaviour of human malignant melanomas. But many human tumours appear to have a multifocal origin or, at least, to develop in a field of abnormal, possibly initiated, cells, which suggests that they may have been pleoclonal initially, even when only one clone has survived the dual hazards of host defence and interclonal competition. Moreover, even when the multistage journey to malignant transformation is completed by only one cell, there is still the possible generation of diversity within this clone by mutation or epigenetic change, and subsequent selection. The simultaneous regression of some melanoma metastases and progression of others which, as I mentioned earlier, was first reported by Bodenham and the recent observation of Albino *et al.* (1981) that 3 established lines of melanoma cells from different metastases in the same patient showed differences in growth rate, morphology, pigmentation, and expression of surface antigens and glycoproteins, suggest that this phenomenon occurs in human tumours, as well as in tumours of experimental animals.

*A personal view.*—Let me end on a personal note. One of my colleagues, in a recent seminar, quoted a remark which she attributed to Gordon Sato, to the effect that one must have a prejudice when attacking a scientific problem. I think there is much truth in this anti-Popperian aphorism, though I would prefer the word *conviction* to *prejudice.* Karl Popper (1968) performed a great service for science by his forceful reiteration of the need to test and retest our hypotheses experimentally. But if all our energy is devoted to trying to disprove current hypotheses we shall have no energy left to generate new ones. Our understanding of cosmology and gravitation was advanced, not impeded, by Galileo's conviction that the earth *does* move, and the development of genetics was advanced by Darwin's convictions about the origin of species, though many of their ideas on these subjects have been superseded. It is, therefore, not wrong but necessary in science to have convictions; what is wrong is to neglect to test them, or to maintain them in the face of evidence to the contrary.

I remain convinced that the concept of surveillance in relation to cancer is soundly based, and that advances in the prevention and treatment of cancer will stem from a deeper understanding of the complex interaction of cancer and host; but, who knows, I may be wrong.

I am deeply indebted to Professor H. J. Evans for the privelege of working in his Unit, and to the Medical Research Council for a Project Grant. I am also grateful to the many colleagues I have named for their collaboration and for allowing me to quote the results of joint work and to Grune & Stratton Inc. for permission to quote freely from my book, *The Interaction of Cancer and Host—Its Therapeutic Significance.*

### REFERENCES

ALBINO, A. P., LLOYD, K. O., HOUGHTON, A. N., OETTGEN, H. F. & OLD, L. J. (1981) Heterogeneity in surface antigen and glycoprotein expression of cell lines derived from different melanoma metastases of the same patient. Implications for the study of tumor antigens. *J. Exp. Med.*, **154**, 1764.

COCHRAN, A. J., ARGOV, S., KÄRRE, K., KLEIN, G. O. & KLEIN, G. (1982) Incidence and type of tumours induced by oral DMBA in NK-deficient C57BL BG/BG mice, +/BG littermates and H-2 congenic strains of B10 of varying NK activity. *Br. J. Cancer*, (BACR abst.), **46**, this issue.

HART, I. R. (1982) The development of metastatic heterogeneity in malignant tumours. *Br. J. Cancer*, (BACR abst.), **46**, 514.

HOLDEN, H. T. (1982) Diversity of anti-tumour effector mechanisms. BACR 23rd A.G.M., unpublished.

KRIPKE, M. L. (1981) Immunologic mechanisms in UV radiation carcinogenesis. *Adv. Cancer Res.*, **34**, 69.

MCBRIDE, W. H., WOODRUFF, M. F. A., FORBES, G. F. & MOORE, K. (1982) Effect of *C. parvum* on the number and activity of macrophages in primary and transplanted murine fibrosarcomas. *Br. J. Cancer*, **46**, 448.

MELICOW, M. M. (1982) The three steps to cancer: a new concept of carcinogenesis. *J. Theor. Biol.* **44**, 471.

POPPER, K. R. (1968) *The Logic of Scientific Discovery.* (revised edn). London: Hutchinson.

POSTE, G., DOLL, J. & FIDLER, I. J. (1981) Interactions among clonal subpopulations affect stability of the metastatic phenotype in polyclonal populations of B16 melanoma cells. *Proc.. Natl. Acad. Sci.*, **78**, 6226.

PREHN, R. T. & OUTZEN, H. C. (1980) Immunostimulation of tumour growth. In *Prog. in Immunology IV, Immunology* '80 (Eds. Fougereau & Dausset). London: Academic Press, p. 651.

SALMON, S. (1982) Biological and clinical studies of clonogenic human tumour cells. *Br. J. Cancer*, (BACR abst.), **46**, 460.

STEEL, G. G., COURTENAY, V. D. & ROSTOM, A. Y. (1978) Improved immune-suppression technique for the xenografting of human tumours. *Br. J. Cancer*, **37**, 224.

STUTMAN, O. (1982) Natural cell-mediated cytotoxicity as a possible anti-tumour surveillance mechanism. *Br. J. Cancer* (BACR abst.), **46**, 461.

WHEELOCK, E. F., WEINHOLD, K. J. & LEVICH, J. (1981) The tumor dormant state. *Adv. Cancer Res.*, **34**, 107.

WOODRUFF, M. F. A. (1980) *The Interaction of Cancer and Host: Its Therapeutic Significance.* New York: Grune & Stratton.

WOODRUFF, M. F. A., ANSELL, J. D., FORBES, G. M., GORDON, J. C., BURTON, D. I. & MICKLEM, H. S. (1982*a*) Clonal Interaction in tumours. *Nature*, in press.

WOODRUFF, M. F. A., FORBES, G. F. & GORDON, J. (1982*b*) Immunogenicity, macrophage sensitivity and therapeutic response to *C. parvum* of fibrosarcomas induced in *C. parvum*-treated and untreated mice. *Cancer Immunol. Immunother.* **12**, 255.

WOODRUFF, M. F. A., FORBES, G. F. & SPEEDY, G. (1982*c*) Further studies on the inhibition of chemical carcinogenesis by *Corynebacterium parvum. Cancer Immunol. Immunother.* **12**, 259.

Papers cited in the text and published prior to 1981, which are not listed above will be found in Woodruff (1980).